# Recent Advances in Quantitative Nuclear Medicine and Molecular Imaging

**DOI:** 10.1155/2018/3613156

**Published:** 2018-06-25

**Authors:** Yun Zhou, Jie Lu, Chongzhao Ran, Jianhua Zhang

**Affiliations:** ^1^The Russell H. Morgan Department of Radiology and Radiological Science, Johns Hopkins University School of Medicine, Baltimore, MD 21287, USA; ^2^Department of Radiology, Xuanwu Hospital, Capital Medical University, Beijing, China; ^3^Martinos Center for Biomedical Imaging, Harvard Medical School, Boston, MA 02129, USA; ^4^Department of Nuclear Medicine, Peking University First Hospital, Beijing, China

Whole-body PET-CT, simultaneous whole-body PET-MRI, and multimodal molecular imaging system are the recent milestone development in nuclear medicine and molecular imaging. These high-end molecular imaging scanners with probes or tracers provide the potential to significantly improve the accuracy in localization and quantification of biological processes at cellular and molecular levels in humans and other living systems. However, there are many challenges in image processing and mathematical and statistical modeling to extract physiological and biochemical information from these multimodal image data. Tremendous efforts have been made recently in the evaluation of the advanced imaging system, extracting physiological and biochemical parameters from multimodal images, integration of multiparametric images, and exploring the applications of the advanced quantitative molecular imaging in clinical nuclear medicine and biology. This special issue invited a number of papers to update the recent advances by those investigators in quantitative nuclear medicine and molecular imaging.

Reconstruction methods play a vital role for the quality and quantification of PET imaging. Time of flight (TOF) and point spread function (PSF) with ordered subset expectation maximization (OSEM) are implemented in most advanced PET-CT and PET-MRI scanners to improve image quality. In the first paper, the effects of image reconstruction algorithms on the kinetic modeling of ^11^C-acetate PET imaging for myocardium assessment were studied. The study found that the image quality from OSEM/TOF/(TOF + PSF) was significantly better than that from the classical image reconstruction method with filtered backprojection.

To extract multiphysiological or biochemical information from multimodal image data and integrate with the mathematical and statistical model is a key to improve the clinical diagnosis accuracy. In the second paper, the combination of ^68^Ga-DOTATATE and ^18^F-FDG could provide better clinical and prognostic evaluation for gastroenteropancreatic neuroendocrine neoplasms (GEP-NENs). They found that the sensitivity of PET/CT imaging with the dual tracers was well associated with cell differentiation, as evidenced by a significant correlation between Ki-67 and SUVmax of PET-CT with both tracers. In the third paper, the researchers assessed the prognostic value of standardized uptake value (SUV), whole-body metabolic tumor volume (WBMTV), and whole-body total lesion glycolysis (WBTLG) for patients with nasopharyngeal carcinoma (NPC) after therapy using FDG PET-CT. Receiver operating characteristic curves were used for the evaluation. They concluded that WBMTV could be used to improve the detection rate of metastatic lesions, and WBTLG could be used as an independent prognostic indicator for NPC after therapy. In the eighth paper, the researchers use ^18^F-FDG PET, diffusion-weighted imaging (DWI), and dynamic contrast-enhanced (DCE) imaging to study the relationship among the quantitative measurements of tumor ^18^F-FDG SUV, apparent diffusion coefficient (ADC), volume transfer constant *k*_trans_, volume of the extravascular extracellular leakage space *V*_e_, and the parameter *k*_e_ for diffusion of the contrast medium from the EES back to the plasma, for grading head and neck squamous cell carcinoma (HNSCC). They found that the associations among those parameters were dependent on the stages of HNSCC. The results provide the potential to improve the accuracy of HNSCC grading. In the ninth article, a novel quantitative parameter, the stress-to-rest ratio of the signal-to-noise ratio (RSNR), was proposed to quantify the myocardial perfusion image generated from a cadmium-zinc-telluride (CZT) single-photon emission computed tomography (SPECT) scanner. Results from the study demonstrated that the RSNR was a useful index to assist the diagnosis of triple-vessel disease.

Now, more and more physicians and scientists are interested to use artificial intelligence technologies such as deep learning for assistance in medical image-based diagnosis. In the sixth paper, the authors proposed to use two convolutional neural networks (CNNs), V-Net and W-Net, for lesion detection on ^68^Ga-Pentixafor PET/CT images. The deep learning method was evaluated by phantom-based simulation data and multiple myeloma ^68^Ga-Pentixafor PET/CT patient study. Traditional machine learning methods for tumor detection including random forest classifier (RF), k-nearest neighbors (k-NN), and support vector machine (SVM) were also applied to the same data sets for comparison. Results from the study showed that the proposed deep learning method with PET and CT provided highest lesion detection accuracy.

Functional MRI of the brain's (FMRIB's) linked independent component analysis (FLICA) is a data-driven approach for multimodal image data analysis. The authors in the fifth paper applied FLICA to FDG PET images collected from Huashan Hospital and multicenter Alzheimer's Disease Neuroimaging Initiative (ADNI) studies. They found that some brain regions were negatively correlated with age, and the correlations between those brain regional SUVRs and 3 clinical assessments were significantly improved if age correction was applied to SUVRs.

In the past years, brown adipose tissue (BAT) has been considered as an important and potential therapeutic target for obesity and diabetes. Reportedly, results of BAT imaging can be varied under different conditions such as cold exposure and thyroid hormone levels. In this special issue, two groups have reported their results from preclinical mouse imaging or human imaging. The authors in the seventh paper showed that spectral unmixing imaging with a near-infrared fluorescent probe could be used to dissect the imaging signal from BAT activation and BAT mass. In addition, the authors in the fourth paper showed that elevated TSH condition before RIT, hyperthyroidism, or hypothyroidism had no significant impact on BAT visualization with FDG PET-CT imaging.

The researchers in the tenth paper previously demonstrated that ^111^In-labeled autologous leukocyte scintigraphy could be used to detect osteomyelitis in living juvenile pigs. To further confirm the exact location and better image interpretation, imaging after euthanasia is always necessary. In this paper, they indicated that SPECT/CT with the same imaging probe at 24 hours after euthanasia could provide the same detectability of osteomyelytic lesions as in living pigs.

*Yun Zhou* *Jie Lu* *Chongzhao Ran* *Jianhua Zhang*

